# Pediatric Ankle Fractures: Successful Remodeling and Restoration Through Comprehensive Diagnosis and Conservative Management in a Diverse Context

**DOI:** 10.7759/cureus.53547

**Published:** 2024-02-04

**Authors:** Evmorfia Pechlivanidou, Orestis Constantas, Evangelos Kallaras, Alexandros Chatzikyriakos, Rodanthi Margariti, Nikolaos Sekouris, Panteleimon N Zogakis

**Affiliations:** 1 1st Department of Orthopedics, P. & A. Kyriakou Children's Hospital, Athens, GRC; 2 Department of Hygiene, Epidemiology, and Medical Statistics, National and Kapodistrian University of Athens School of Medicine, Athens, GRC

**Keywords:** comprehensive diagnostic approach, socioeconomic considerations, conservative management, multifaceted fractures, pediatric ankle fractures

## Abstract

Diagnosing toddler ankle fractures, especially those that affect several bones, can be difficult. The infrequency of such complex injuries, particularly in household environments, emphasizes the importance of increased awareness in diagnosing and managing these types of injuries.

We present a compelling case study of a 20-month-old toddler of a low socioeconomic background who sustained fractures in the ankle, calcaneus, tibia, and fibula after being trapped under furniture. The diagnostic process involved trauma guidelines, radiographic assessments, and axial CT scans. Conservative management, including an eight-week plaster cast, was chosen based on the careful consideration of the child's age, the nature of the fracture, and the absence of immediate surgical indications. The follow-up period involved radiographic assessments, as well as repeated regular clinical examinations, revealing consistent alignment and the absence of complications.

The successful outcome underscores the importance of a comprehensive diagnostic approach, thoughtful treatment planning, and meticulous follow-up. Individualized care, considering both clinical and socioeconomic factors, proved crucial for optimal outcomes in pediatric orthopedics. The case contributes valuable insights into the evolving landscape of early childhood orthopedics, emphasizing the need for a discerning approach to diagnosing and managing complex fractures in this population. Conservative treatment could significantly assist when absolute surgical indications are lacking both in cases of minimal resources where multiple operations are not plausible and when the patient's social history raises awareness.

## Introduction

In the sphere of pediatric orthopedics, pediatric ankle fractures, though relatively rare, pose distinctive challenges [1]. These fractures, often associated with increased activity levels and participation in sports, predominantly affect older children, typically above the age of six [1-4]. Although more prevalent in males, their occurrence is contingent upon various factors, including age, gender, and activity patterns [1].

However, the incidence of ankle fractures becomes even more intriguing when considering the coexistence of fractures in adjacent bones within domestic environments [5]. Such occurrences, involving the calcaneus, tibia, and fibula simultaneously, are exceptionally rare and typically associated with high-energy trauma, such as crushing injuries [6]. Domestic scenarios, traditionally perceived as lower-risk environments, can unexpectedly harbor complex fractures, particularly when involving toddlers and young children [7]. Their exploratory nature and interaction with the household environment may contribute to unique injury patterns [8,9].

Owing to their scarcity, the diagnosis of pediatric ankle fractures mandates discernment, necessitating a nuanced differentiation between accidental trauma, potential abuse, or alternative non-malicious etiologies [10,11]. Clinicians frequently encounter fractures attributed to the exuberant proclivities inherent in childhood activities. The elucidation of diagnostic intricacies is facilitated by the congruence among the reported incident; clinical presentation characterized by edema, pain, and constrained mobility; and radiological corroboration aligning with anticipated outcomes. The complexity is further compounded when the suspicion of child abuse arises, marked by injuries bereft of plausible explanations or exhibiting incongruities [11]. Classic metaphyseal lesions or atypical fracture configurations may serve as harbingers of potential abuse, warranting a comprehensive investigative appraisal.

In addressing the intricate management and therapeutic modalities applicable to ankle and calcaneus fractures in young children, a comprehensive and tailored approach is imperative. The initial assessment, encompassing scrupulous clinical evaluation and radiographic scrutiny, forms the fulcrum for precise diagnosis [1,12]. Conservative modalities, including casting and non-weight-bearing protocols, constitute the foundational tenets for the management of nondisplaced or minimally displaced fractures, affording stability conducive to natural convalescence [13]. Subsequent vigilance, comprising periodic radiographic assessments and clinical evaluations, ensures the sustained appropriateness of the therapeutic schema. Surgical recourse, specifically embracing open reduction and internal fixation (ORIF), assumes relevance in the context of displaced or intricate fractures [14-16]. Beyond the acute phase, rehabilitative endeavors via physical therapy, embracing targeted exercises and gait restoration, aspire to reestablish optimal functionality [17]. Pain management, patient and familial enlightenment emphasizing activity constraints, and follow-up adherence, alongside longitudinal monitoring accentuating growth dynamics, consummate this sophisticated and interdisciplinary approach [1,7,12].

The following case report describes a complex and detailed case, explaining a difficult situation involving a toddler who was admitted to our emergency department. The child sustained a complex fracture including the ankle, calcaneus, tibia, and fibula as a result of getting trapped under a heavy piece of furniture within the domestic milieu.

## Case presentation

We present a case of a male toddler, aged 20 months, from a low-socioeconomic status (SES) household who presented at the emergency room with a chief complaint of pain in the left lower extremity accompanied by a noticeable limp. The historical account of the child said that shortly prior to seeking medical attention, (approximately half an hour), the little child had become trapped by a huge oak wardrobe within their residence. According to the parents, the wardrobe was inadequately secured in an upright position. While the child was trying to retrieve something from the wardrobe for play, the furniture toppled over, resulting in the toddler's foot becoming trapped below it.

During the examination, the patient exhibited signs of discomfort, and a thorough clinical assessment revealed that the left ankle exhibited tenderness, edema, and an evident gait abnormality. More specifically, the patient was unable to use his left lower extremity adequately, as he had for the previous six months since he began walking. In fact, he was dragging the affected leg while walking. The initial assessment did not reveal any other significant injuries or abnormalities.

**Figure 1:**
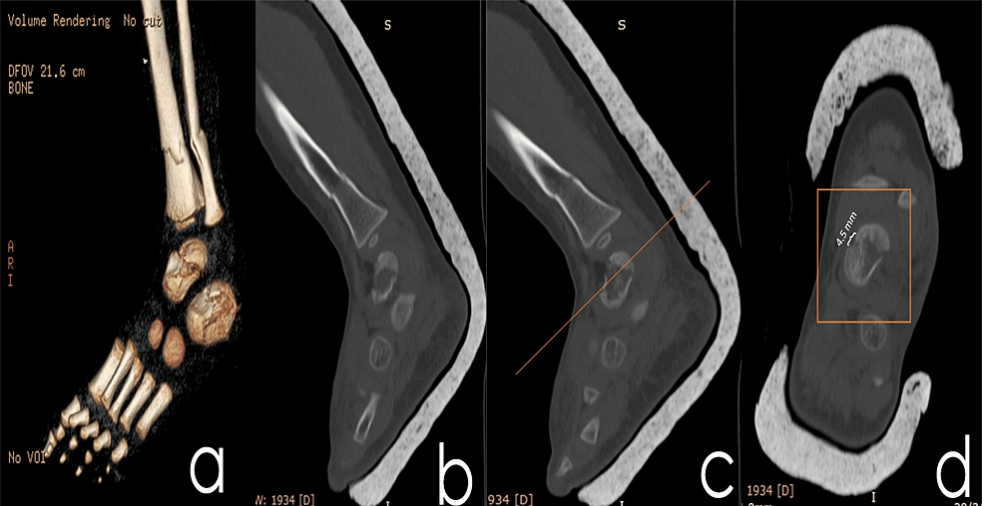
The multiple fractures of the case, as depicted at the CT, performed on the first day the patient presented at the emergency department (a) 3D reconstructive imaging; (b and c) sagittal views showing tibial, talus, and calcaneus fractures; and (d) axial view of the talus fracture showing 4.5 mm displacement in the talus fracture

A radiograph of the damaged area was promptly obtained in accordance with the trauma guidelines implemented at the facility. The radiographic examination demonstrated multiple fractures, which included a Hawkins type I comminuted displaced fracture of the neck of the talus; an intra-articular, Sanders type I fracture of the calcaneus; and a diaphyseal fracture involving both the tibia and the fibula. The subsequent axial CT scan yielded a precise measurement of a 4.5 mm displacement in the talus fracture, thus corroborating the earlier observations and furnishing further details regarding the nature of the fracture (Figure 1).

After exhaustively eliminating other potential contributing factors, such as instances of mistreatment or pathological disorders, a prudent choice was reached to adopt a careful strategy in handling the circumstances. The individual, hailing from a disadvantaged socioeconomic status (in case of surgical intervention, hospitalization would be long in order to prevent infection and miscarriage due to domestic circumstances), was rendered immobile with the application of a plaster cast; a long leg cast (10 cm SafeCast® plaster cast, MEDICHAIN Medical Devices, Athens, Greece) was used, applied with the knee slightly bent and 90-degree dorsiflexion extending below the knee. As part of the therapy regimen, the patient's parents were instructed that the patient should keep the cast for a duration of eight weeks and remain non-weight-bearing. The decision to employ conservative treatment options for the child's injury was based on several factors, including the child's age, the type of fractures sustained, and the lack of any indications for immediate surgical intervention.

The patient and their family adhered to the immobilizing cast therapy, and signs of cast corrosion were observed during the follow-up. Despite the patient's young age and the inherent propensity for mobility in this particular age group, no concerns arose regarding the integrity of the cast.

**Figure 2:**
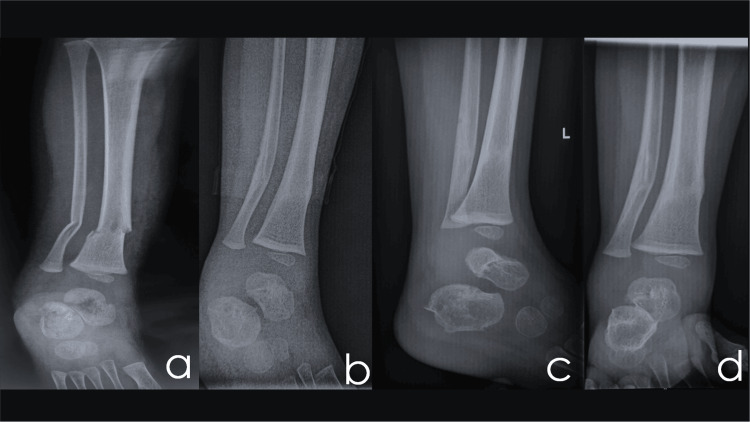
Radiographic course during follow-ups (a) X-ray (face) at the diagnosis, (b) X-ray (face) at one week, (c) X-ray (profile) at three weeks, and (d) X-ray (face) at three weeks

The patient underwent plain radiographs at specified intervals of three days, one week, one month, and subsequently on a monthly basis as per the predetermined follow-up protocol (Figure 2). The last follow-up of the patient was six months after the injury to evaluate possible gait abnormalities.

Throughout the duration of the follow-up period, it was seen that each fracture maintained its alignment at a satisfactory level. Consistently, the radiographs and Hawking's sign suggested the presence of ongoing blood circulation to the talus. No evidence of avascular necrosis or any other complications associated with the fracture was observed.

The toddler promptly resumed his regular daily routines and exhibited rapid physical mobility subsequent to the conclusion of the eight-week period of immobility. At follow-up visits, the child's parents did not express any worries, and the toddler returned back to normal. During the subsequent visits, the parents of the child did not articulate any concerns, and the youngster exhibited a restoration of his prior level of functionality level. Despite the immobilization at the end of the follow-up, the child had regained potential similar to a healthy leg. At the last follow-up, the range of motion (ROM) was fully recovered without restrictions in the sagittal plane, dorsiflexion, and plantarflexion. The ROM was within the normal range for all aforementioned axes, and gait abnormalities were not observed.

## Discussion

This study examines a case that reveals the complexities of toddler ankle fractures occurring simultaneously with fractures in the calcaneus, tibia, and fibula. The rarity of such multifaceted injuries, especially within a domestic setting, underscores the need for heightened awareness and vigilance in diagnosing and managing these cases [1]. By exploring the incidence of pediatric ankle fractures and the infrequent coexistence of fractures in domestic environments, this study contributes valuable insights into the evolving landscape of early childhood orthopedics [2,3]. Through a detailed examination of this unique case, we aim to enhance our understanding of the diagnostic challenges, treatment strategies, and outcomes associated with complex fractures in pediatric orthopedics.

Pediatric ankle fractures, particularly those involving multiple bones simultaneously, present diagnostic challenges that necessitate a comprehensive and discerning approach [7,14]. The rarity of such occurrences, compounded by the diversity of potential injury mechanisms, underscores the importance of meticulous clinical evaluation and thorough radiographic evaluation. In this case, the fractures involved a comminuted displaced talus fracture, calcaneus fracture, and diaphyseal fractures of both the tibia and the fibula. The utilization of trauma guidelines and additional imaging through axial CT scans played a crucial role in achieving a precise diagnosis [5,8,15,17]. Given the potential for severe injuries and considering the age of the patient, a swift and accurate diagnosis was essential for initiating timely and appropriate management.

The decision to pursue conservative management, including immobilization with a plaster cast for eight weeks, reflects a careful consideration of multiple factors [2,10,15]. The child's age, the nature of the fractures, and the absence of immediate surgical indications were pivotal in choosing this approach.

The therapeutic plan for the toddler in this case was intricately shaped by the unique challenges presented by his age group. Toddlers, being in a phase of continuous bone development and growth potential, pose distinct hurdles, including issues related to immobilization compliance [1,5,12]. Opting for conservative care, specifically an eight-week plaster cast, was a deliberate choice considering the ongoing growth of the child's bones. The decision to avoid surgery was rooted in the understanding that surgical interventions might disrupt the natural growth process, a critical factor in the toddler age group [2,10,15]. Recognizing the remarkable capacity of young children for bone repair and reshaping, the conservative approach aimed to harness the inherent healing potential without resorting to more invasive measures. On the contrary, if the child was older, the considerations for surgery may be the main case. Surgical intervention is more likely in infants with ankle fractures with significant displacement causing long-term functional effects [10,16]. The restoration of anatomical alignment becomes the main goal, when conservative approaches risk malunion or functional damage. Open reduction and internal fixation (ORIF) with screws or plates can produce precise and stable results. ORIF is an absolute necessity for adolescents when skeletal maturity is the factor influencing the treatment decision [1]. The exact calendar timing of fractures during the adolescent growth spurt determines surgical fixing. Epiphyseal fractures near growth plates may require precise surgery to avoid growth interruption. Surgical stabilization may improve long-term functional results for teens with high-impact activities or severe fractures.

All these problematics are not parts of the puzzle regarding young toddlers, for whom conservative treatment can be a successful decision for a couple of reasons. First and foremost, toddlers possess growth plates at the ends of their bones that are still in the developmental stage. Surgical interventions near these growth plates carry the risk of disrupting normal bone growth, making conservative measures essential to minimize the potential for growth disturbances [3,14,17]. Additionally, the remarkable bone remodeling ability of young children, including toddlers, allows for natural realignment and healing, obviating the immediate need for surgical intervention [18]. By opting for nonsurgical methods, there is a reduction in the inherent risks associated with anesthesia, which can be challenging in very young children. Furthermore, ankle fractures in toddlers are often less severe, with the bones exhibiting less displacement, making it more feasible to achieve proper alignment through conservative means [10]. The practicality of immobilizing the ankle with a cast is also a factor, ensuring better compliance from both the toddler and their caregivers [3,14,17]. Lastly, the avoidance of surgical risks, including infection, bleeding, and complications related to anesthesia, underscores the comprehensive rationale for choosing conservative treatment whenever possible in the context of ankle fractures in toddlers [1]. Moreover, the long-term impact of surgery on the child's mental health and future growth trajectory was carefully weighed in light of their age.

To sum it up, conservative modalities are foundational in managing nondisplaced or minimally displaced fractures, providing stability conducive to natural healing [10,16]. In this case, the successful adherence to the immobilization therapy by the patient and their family, despite the inherent mobility of the toddler age group, contributed significantly to the positive outcomes observed during follow-up.

The established follow-up protocol, involving radiographic assessments at specific intervals, served as a vital tool for monitoring the healing progress and alignment of fractures. The absence of complications such as avascular necrosis and the maintenance of blood circulation to the talus were encouraging findings. The child's swift return to normal activities and the restoration of pre-injury functionality attest to the efficacy of the chosen therapeutic approach [5,12]. The consistent alignment of fractures throughout the follow-up period further supports the decision for conservative management [1,5,12].

The socioeconomic context of the patient adds a layer of complexity to the therapeutic decision-making process. A household characterized by a low socioeconomic status may entail limitations in access to healthcare resources [4,9]. The decision to opt for a conservative approach, which often aligns with practical considerations, proved effective in this case. This emphasizes the significance of customizing treatment techniques for complex situations, as there is a lack of definitive criteria for surgery and other factors that need to be considered.

Despite the valuable insights provided by this case report, it is essential to acknowledge certain limitations inherent to the study. Firstly, the report represents a single case study, limiting the generalizability of findings to a broader population. The uniqueness of the presented scenario, involving a toddler trapped under furniture with multiple fractures, may not fully encapsulate the diverse spectrum of pediatric ankle fractures. Additionally, the retrospective nature of the study design introduces the potential for recall bias and dependence on available medical records. The lack of a comparative group further restricts the ability to draw direct comparisons between different management approaches. Furthermore, the study does not delve into the psychosocial aspects and long-term impact on the child's quality of life, which could offer a more comprehensive understanding of the holistic implications of such injuries. Future research endeavors with larger sample sizes, prospective designs, and a more comprehensive assessment of outcomes will be crucial to addressing these limitations and advancing our understanding of pediatric ankle fractures in diverse contexts.

## Conclusions

In conclusion, this case emphasizes the multifaceted nature of pediatric orthopedic management, especially in scenarios involving rare and complex fractures. The successful outcome in this case is attributed to a comprehensive diagnostic approach, thoughtful treatment planning, and meticulous follow-up. It underscores the importance of individualized care, considering the complexity of all these coexistent fractures, the lack of a clear surgical indication, the growth and remodeling potential of the patient, and other factors related to the patient's care, to achieve optimal outcomes in pediatric orthopedics. It underscores the importance of individualized care, considering both clinical and socioeconomic factors, to achieve optimal outcomes in pediatric orthopedics. Such an approach could be even more beneficial for areas with limited resources where optimal outcomes could be combined with limited intervention.
